# Effect of an Emergency Department Fast Track on Press-Ganey Patient Satisfaction Scores

**DOI:** 10.5811/westjem.2014.11.21768

**Published:** 2014-12-05

**Authors:** Calvin E. Hwang, Grant S. Lipman, Marlena Kane

**Affiliations:** * Stanford University School of Medicine, Stanford/Kaiser Emergency Medicine Residency, Stanford, California; † Stanford University School of Medicine, Division of Emergency Medicine, Department of Surgery, Stanford, California; ‡ Stanford Hospital and Clinics, Department of Patient Care Services, Stanford, California

## Abstract

**Introduction:**

Mandated patient surveys have become an integral part of Medicare remuneration, putting hundreds of millions of dollars in funding at risk. The Centers for Medicare & Medicaid Services (CMS) recently announced a patient experience survey for the emergency department (ED). Development of an ED Fast Track, where lower acuity patients are rapidly seen, has been shown to improve many of the metrics that CMS examines. This is the first study examining if ED Fast Track implementation affects Press-Ganey scores of patient satisfaction.

**Methods:**

We analyzed returned Press-Ganey questionnaires from all ESI 4 and 5 patients seen 11AM – 1PM, August–December 2011 (pre-fast track), and during the identical hours of fast track, August–December 2012. Raw ordinal scores were converted to continuous scores for paired student t-test analysis. We calculated an odds ratio with 100% satisfaction considered a positive response.

**Results:**

An academic ED with 52,000 annual visits had 140 pre-fast track and 85 fast track respondents. Implementation of a fast track significantly increased patient satisfaction with the following: wait times (68% satisfaction to 88%, OR 4.13, 95% CI [2.32–7.33]), doctor courtesy (90% to 95%, OR 1.97, 95% CI [1.04–3.73]), nurse courtesy (87% to 95%, OR 2.75, 95% CI [1.46–5.15]), pain control (79% to 87%, OR 2.13, 95% CI [1.16–3.92]), likelihood to recommend (81% to 90%, OR 2.62, 95% CI [1.42–4.83]), staff caring (82% to 91%, OR 2.82, 95% CI [1.54–5.19]), and staying informed about delays (66% to 83%, OR 3.00, 95% CI [1.65–5.44]).

**Conclusion:**

Implementation of an ED Fast Track more than doubled the odds of significant improvements in Press-Ganey patient satisfaction metrics and may play an important role in improving ED performance on CMS benchmarks.

## INTRODUCTION

In October 2012, the Centers for Medicare & Medicaid Services (CMS) announced a new hospital-payment system called value-based purchasing (VBP). This initiative tied 964 million dollars of federal hospital reimbursement in its first year of implementation to a combination of clinical process of care and patient experience of care domains,^1^ with the former comprising 70% and the latter 30% of the overall score.^2^ The patient experience of care domain in VBP is based on the Hospital Consumer Assessment of Healthcare Providers and Systems (HCAHPS) survey and encompasses eight aspects of the consumer experience in the healthcare system: communication with nurses, communication with doctors, responsiveness of hospital staff, pain management, cleanliness and quietness of hospital environment, communication about medicines, discharge information, and overall rating of hospital.^2^ Press-Ganey surveys of patient satisfaction are currently employed by almost 50% of the hospitals in America, with many questions in areas directly targeted by the HCAPS survey.^3^ There is increasing hospital awareness of customer satisfaction with implementation of VBP, and some states have linked physician salaries to patient satisfaction.^4^ Moreover, there appears to be a trend amongst emergency physician groups linking compensation and incentive payments to patient satisfaction scores, though no published data on this currently exists in the literature today.

Emergency department (ED) fast track is a designated area where lower acuity ED patients are rapidly seen. ED Fast Tracks have become more prevalent in recent years, with nearly 80% of EDs in the United States currently incorporating some type of fast track area.^5^ ED Fast Track has been shown to improve several metrics associated with both provider and patient satisfaction.^6^,^7^ This is the first study examining if implementation of an ED Fast Track affects Press-Ganey scores of patient satisfaction.

## METHODS

### Study Design and Setting

This was a serial before and after cross-sectional study of Press-Ganey questionnaires completed by low acuity and ED Fast Track patients seen in an academic ED with approximately 50,000 visits annually from August–December 2011 and August–December 2012. The study was deemed exempt by the Stanford University School of Medicine institutional review board.

### Methods, Measurements and Outcomes

Press-Ganey questionnaires were sent to 100% of discharged ED patients with a 4-day lag from the visit date. We analyzed returned surveys were analyzed from all low acuity patients (defined as Emergency Severity Index 4 and 5, e.g. stable patient requiring only one or fewer resources) seen 11AM–11PM, August–December 2011 (pre-Fast Track) and during the identical hours of ED Fast Track August–December 2012. The medical record numbers on the Press-Ganey file were linked to an ED Arrival Flat File to ensure that multiple patient visits were matched with the correct survey. A review was performed to ensure the dates of service matched. We selected for analysis survey data for seven areas corresponding to the patient experience of care: wait times, nurse courtesy, doctor courtesy, being kept informed about delays, staff caring, pain control, and likelihood to recommend.

### Intervention

A new ED Fast Track was created in July 2012, operating from 11AM–11PM daily for low acuity patients and staffed with its own attending physician, nurse, and ED technician. This required the addition of 2.4 full time equivalents (FTEs) to the ED attending staff. No mid-level providers or residents were used. The patient care area consisted of three chairs in one large room that was newly allocated to the ED from another department at the start of the study period. Radiographs and intravenous medications could be administered in fast track, while any computed tomography or more advanced imaging would be done in radiology. All fast track staff were part of the larger ED pool and were randomly assigned to the fast track area. Prior to implementation of the ED Fast Track, no Fast Track type area existed and all patients presenting to the ED were seen in the main department. Patients presenting to the ED during these times with low acuity chief complaints were identified on arrival and immediately routed to the ED Fast Track area.

### Analysis

We then converted raw ordinal Press-Ganey scores for the appropriate ESI 4 and 5 patient visits to continuous scores used to calculate the mean result for each question. The pre-intervention group consisted only of returned surveys from patients with ESI 4 and 5 presenting to the ED during the same time of day as the post-intervention group in order to include the same acuity and type of patient. We subsequently used these data to calculate student t-test scores pre- and post-intervention. The raw ordinal scores were also used to calculate an odds ratio, with only a 100% satisfaction response (represented by a 5 out of 5 response on the Press-Ganey Likert scale) for a particular question considered a positive result. P-values < 0.05 were considered significant. We performed all statistical analyses using MedCalc for Windows (version 12.7.8, Ostend, Belgium).

## RESULTS

**Figure 1:**
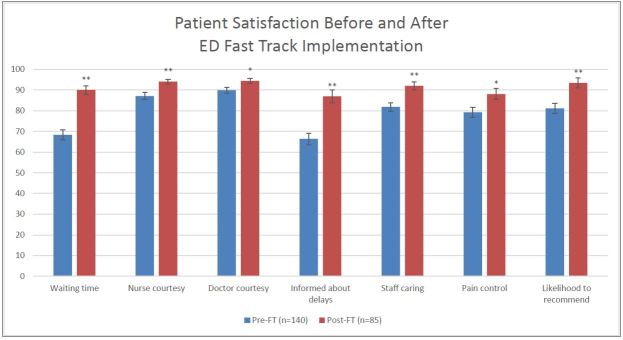
Comparison of patient satisfaction scores before and after implementation of ED Fast Track. *ED*, emergency department ^*^p <0.05; ^**^p <0.01

**Figure 2:**
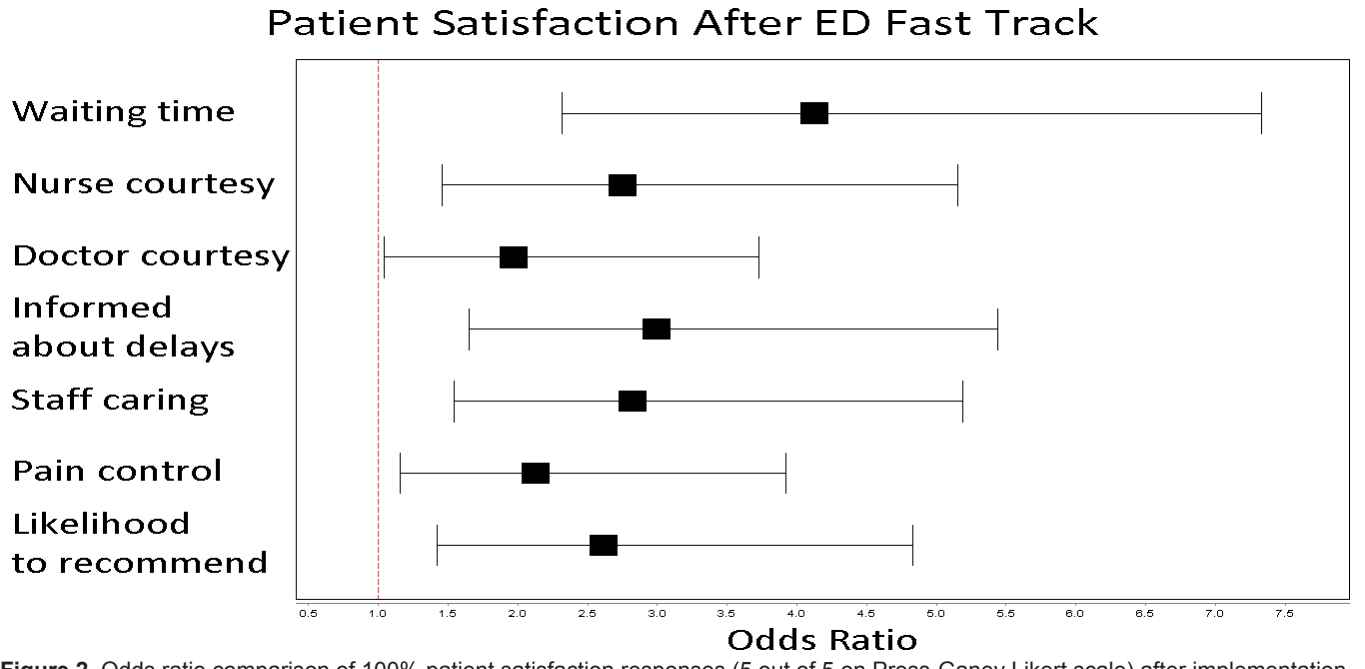
Odds ratio comparison of 100% patient satisfaction responses (5 out of 5 on Press-Ganey Likert scale) after implementation of ED fast track. *ED*, emergency department

We analyzed 140 respondents in the pre-ED Fast Track group and 85 in the ED Fast Track group, with an overall 14.8% response rate. Patients in the pre-ED Fast Track and ED Fast Track cohort represented approximately 9% of the overall ED volume during each time period. There were significant improvements in patient satisfaction after the implementation of an ED Fast Track area in each of the seven categories selected for analysis (Figures 1 and 2). Patient satisfaction with wait times increased from 68% to 88% (OR 4.13, 95% CI [2.32–7.33], p<0.0001), doctor courtesy 90% to 95% (OR 1.97, 95% CI [1.04–3.73], p=0.05), nurse courtesy 87% to 95% (OR 2.75, 95% CI [1.46–5.15], p<0.01), staying informed about delays 66% to 83% (OR 3.00, 95% CI [1.65–5.44], p<0.0001), staff caring 82% to 91% (OR 2.82, 95% CI [1.54–5.19], p<0.01), pain control 79% to 87% (OR 2.13, 95% CI [1.16–3.92], p=0.018), and likelihood to recommend 81% to 90% (OR 2.62, 95% CI [1.42–4.83], p<0.01).

## DISCUSSION

This is the first study to the authors’ knowledge that demonstrated a statistically significant improvement in patient satisfaction with the implementation of an ED Fast Track. While prior studies examined improvements in time metrics,^6^ these are indirectly linked to satisfaction.^7^ Our study relied on a cross sectional survey of patients’ perspectives with a response rate of approximately 15%, similar to national average of returns for surveys of this type.^8^ The determinants of VBP will be based on surveys that will have response rates similar to our study.

In 2008, the first national, standardized, publicly reported patient experience of care survey for the inpatient hospital experience was implemented (Hospital Consumer Assessment of Healthcare Providers and Systems – HCAHPS).^2^ Until this survey, there was no national standard for collecting and publicly reporting information about the patient experience that supported comparisons between hospitals. Beginning in October 2012, hospitals that performed poorly on these measures had to forfeit a percentage of their Medicare payments through the new Hospital Value-Based Purchasing Program. However, this survey does not address a patient’s ED experience. The Centers for Medicare & Medicaid Services (CMS) and the Agency for Healthcare Research and Quality (AHRQ) have recently announced that a patient experience-of-care survey for the ED is next in line (ED CAHPS), given that the ED is often considered the “front door” to the hospital and is an essential component to patients’ overall hospital experience. This tool will survey patients and caregivers of patients who have received care in an ED to evaluate items such as “waiting time to see physician” and “communication with providers.” In addition, in January 2012 CMS began monitoring median time between ED arrival and when the patient leaves the ED to an inpatient room and ED median time from ED admit decision to when the patient leaves the ED to an inpatient room. Following past CMS practices, these metrics will likely be factored into hospital reimbursement in the near future.

As healthcare providers face more pressure to manage costs and do more with less, patient satisfaction surveys like Press-Ganey can be an invaluable tool to improve the patient experience, as well as overall operational performance. Collecting patient satisfaction data helps to provide an understanding of potential opportunities for improvement and may prevent organizations from implementing solutions that are not connected to the root cause of the problem. Moreover, understanding and acting on patient concerns will support hospitals in getting ready for the new ED CAHPS quality measures that will be put in place to measure how satisfied patients are with their visit to the ED.

Development of an ED Fast Track has enabled the studied ED to improve the value of care delivered to patients, despite the operational challenges of a growing census and space constraints that are being faced by EDs throughout the nation, ultimately resulting in quicker service, increased capacity, and improved patient satisfaction. A number of patient-centered metrics outside the control of the ED, including a 4% increase in overall volume and a 37% increase in hours spent boarding, worsened in the post-ED Fast Track implementation period. Despite these forces that would normally lead to worse patient satisfaction, not only was implementation of ED Fast Track associated with an improvement in Press-Ganey patient satisfaction scores amongst this cohort of patients, but other patient care metrics for the entire department such as median length of stay and door-to-MD time each also improved by 9%. One hypothesis for this improvement is that the ED Fast Track allows for rapid turnover of low acuity patients, optimizing flow and resources in the rest of the department.

This is timely considering the mandated patient surveys portion of CMS remuneration will soon place hundreds of millions of dollars in federal funding at risk if EDs do not meet performance and quality standards. It remains to be seen how incorporation of an ED Fast Track impacts the care and flow of patients through the other parts of the ED.

## LIMITATIONS

We analyzed a particular subset of low acuity ED patients in this study. Only patients seen and discharged from the ED Fast Track were analyzed in 2012 population. However, the pre-fast track cohort was gathered from their triage index, and it was unknown if they had been under-triaged or would have been fast track appropriate, possibly leading to underestimation of acuity and resulting over-estimation of the intervention’s significance. There was also a noticeable difference in the size of the pre- and post-intervention group, potentially biasing the results. As there is no available database allowing for comparison of individual hospital performances, it is not known if these improvements were part of a generalized trend towards improvement nationwide or indeed unique to this institution. It is also unknown if resource utilization was different in the ED Fast Track, which could have impacted patient satisfaction. However, given that all patients were triaged prior to MD evaluation based on a pre-defined ESI criteria incorporating number of resources anticipated to be used, it is likely that the same types of patients were present in both the pre and post-intervention groups and resource utilization would not be significantly different.

While we could not determine if our observed improvements in patient satisfaction were in part due to decreased lengths of stay, shorter wait times, dedicated resource utilization, or other unknown variables, implementation of an ED Fast Track program was clearly associated with increased patient satisfaction. Nevertheless, further studies need to be done on the cost of ED Fast Track. In this study, a hospital space was reallocated to the ED to serve as the ED Fast Track, and additional staff and physician time had to be allocated. Additional funds from VBP as a result of increased satisfaction and efficiency from the ED Fast Track may outweigh these costs. This is also a potential confounder to the data as the addition of ED space and staffing alone could also have improved these Press-Ganey metrics.

## CONCLUSION

The implementation of an ED Fast Track program was associated with statistically significant improvements in seven dimensions of Press-Ganey patient satisfaction metrics. With the initiation of value-based purchasing and subsequent linkage of hospital reimbursement with the patient care experience, implementation of ED Fast Track programs may play an important role in improving ED performance on CMS benchmarks of quality with lower acuity patients.
